# Complete Genome Sequence of H1N1 Swine Influenza Virus from Pigs in the Republic of Korea in 2016

**DOI:** 10.1128/MRA.01229-18

**Published:** 2018-12-13

**Authors:** Ju-Yeon Lee, In-Ohk Ouh, Soo-Dong Cho, In-Soo Cho, Choi Kyu Park, Jae-Young Song

**Affiliations:** a Viral Disease Division, Animal and Plant Quarantine Agency, Gimcheon, Gyeongsangbuk-do, Republic of Korea; b College of Veterinary Medicine and Animal Disease Intervention Center, Kyungpook National University, Daegu, Republic of Korea; University of Arizona

## Abstract

We report here the genome sequence of the influenza A virus strain A/swine/Korea/61/2016, isolated from swine in the Republic of Korea. On the basis of sequence analysis, A/swine/Korea/61/2016 is marked from swine H1N1 influenza virus.

## ANNOUNCEMENT

Swine influenza A virus (H1N1) was derived from several viruses circulating in swine, and the initial transmission to humans occurred several months before recognition of the outbreak (1). Genetic reassortment in pigs allows for the generation of novel influenza viruses and further demonstrates that pigs can serve as intermediate hosts and act as “mixing vessels” for human, swine, and avian influenza viruses (2). To date, mortality associated with pandemic A/H1N1 2009 influenza has been reported with variable completeness worldwide (3) and in particular subgroups, including inpatients (4), patients in critical care (5–7), pregnant women (8), and children (9).

Here, we report on a complete sequence of H1N1 influenza virus that was identified in nasal swabs taken from domestic pigs on farms located in Gyeongbuk Province, Republic of Korea, in 2016. H1N1 swine influenza virus (SIV) was isolated in 10-day-old embryonated specific-pathogen-free (SPF) chicken eggs (Valo, USA), and 3 days later, the allantoic fluid was collected. The viral RNAs were extracted from the first passage using an RNeasy minikit (Qiagen, Inc., Germany). Eight viral segments were generated by the SIV isolate using universal influenza primers as protocols as described (10). For the reverse transcription and PCR amplification reactions, a superscript III reverse transcription-PCR (RT-PCR) kit (Invitrogen, USA) was used. The RT-PCR products were ligated to pDrive vectors (Qiagen, Inc.). The recombinant plasmids were isolated from the colonies with a plasmid prep kit (Inclone Biotech, Republic of Korea). The sequences in plasmids were confirmed by DNA sequencing (Macrogen, Republic of Korea). The sequences were analyzed and assembled using DNASTAR version 5.0.

The genome of the A/swine/Korea/61/2016 (H1N1) strain consisted of the following eight gene segments: polymerase (PB2, PB1, and PA), hemagglutinin (HA), nucleoprotein (NP), neuraminidase (NA), matrix protein (M), and nonstructural protein (NS). The sequence of the PB2 gene consisted of 2,341 nucleotides (nt), of the PB1 gene, 2,341 nt, of the PA gene, 2,233 nt, of the HA gene, 1,776 nt, of the NP gene, 1,564 nt, of the NA gene, 1,428 nt, of the M gene, 1,027 nt, and of the NS gene, 890 nt. Phylogenetic analyses showed relatedness of the HA gene to those of the 2009 strains (up to 97% nucleotide identity with the LACENRS-1626 strain) and human isolates, while the NA gene was found to be more similar to the Guangdong human-derived H1N1 SIV strains (97% nucleotide identity).

The pandemic 2016 H1N1 strain is evolving in Republic of Korea pig herds.

### Data availability.

The genome sequences of A/swine/Korea/61/2016 (H1N1) have been deposited in GenBank under the accession numbers MH828297 to MH828304.
